# Identification, Expression, and Functional Analysis of the Group IId WRKY Subfamily in Upland Cotton (*Gossypium hirsutum* L.)

**DOI:** 10.3389/fpls.2018.01684

**Published:** 2018-11-21

**Authors:** Lijiao Gu, Hantao Wang, Hengling Wei, Huiru Sun, Libei Li, Pengyun Chen, Mohammed Elasad, Zhengzheng Su, Chi Zhang, Liang Ma, Congcong Wang, Shuxun Yu

**Affiliations:** ^1^State Key Laboratory of Cotton Biology, Institute of Cotton Research, Chinese Academy of Agricultural Sciences, Anyang, China; ^2^College of Agronomy, Northwest A and F University, Yangling, China

**Keywords:** group IId WRKY genes, expression patterns, stress response, Gh_A11G1801, upland cotton

## Abstract

WRKY transcription factors have diverse functions in regulating stress response, leaf senescence, and plant growth and development. However, knowledge of the group IId WRKY subfamily in cotton is largely absent. This study identified 34 group IId WRKY genes in the *Gossypium hirsutum* genome, and their genomic loci were investigated. Members clustered together in the phylogenetic tree had similar motif compositions and gene structural features, revealing similarity and conservation within group IId WRKY genes. During the evolutionary process, 14 duplicated genes appeared to undergo purification selection. Public RNA-seq data were used to examine the expression patterns of group IId WRKY genes in various tissues and under drought and salt stress conditions. Ten highly expressed genes were identified, and the ten candidate genes revealed distinct expression patterns under drought and salt treatments by qRT-PCR analysis. Among them, Gh_A11G1801 was used for functional characterization. GUS activity was differentially induced by various stresses in *Gh_A11G1801p::GUS* transgenic *Arabidopsis* plants. The virus-induced gene silencing (VIGS) of Gh_A11G1801 resulted in drought sensitivity in cotton plants, which was accompanied by elevated malondialdehyde (MDA) content and reduced catalase (CAT) content. Taken together, these findings obtained in this study provide valuable resources for further studying group IId WRKY genes in cotton. Our results also enrich the gene resources for the genetic improvements of cotton varieties that are suitable for growth in stressful conditions.

## Introduction

In the natural environment, plants are constantly suffering from various adverse external environmental conditions; as a result, plants have evolved a set of adaption strategies to meet these challenges. These adaptive responses cause distinct changes in gene expression that are primarily mediated by transcription factors (TFs) (Singh et al., 2002; Mahalingam et al., 2003; Jang et al., 2010). WRKY proteins constitute a large family of these TFs, and many reports have demonstrated that the WRKY gene family has multiple developmental and physiological functions that are evoked in response to external stimuli (Eulgem et al., 2000; Rushton et al., 2010).

The name of the WRKY gene family is derived from the prominent structural feature of these proteins, the WRKY domain. Proteins in this family contain at least one highly conserved WRKY domain, which includes the conserved WRKYGQK sequence followed by a C2H2- or C2HC-type zinc finger motif in the C-terminal region (Eulgem et al., 2000; Wu et al., 2005). Based on the number of WRKY domains and the types of zinc finger motifs, the WRKY TF family can be subdivided into three primary groups: groups I, II, and III [10]. The group II members, which contain a single WRKY domain and the C2H2-type zinc finger motif, are further categorized into five subgroups, IIa–e, based on phylogenetic analysis and their variation in conserved motifs (Eulgem et al., 2000; Ross et al., 2007; Wang et al., 2011). Phylogeny, sequence comparison and structural analysis provide evidence for the occurrence of gene expansion events among the group IId WRKY genes during evolution [8, 14, 19].

With the completion of whole-genome sequencing, increasing numbers of corresponding group IId WRKY gene members have been recognized genome wide and characterized in different plant species (Li et al., 2017a). As summarized by Li et al., approximately 7 representative group IId WRKY members have been found in *Sesamum indicum*, 8 in *Cucumis sativus*, 7 in *Arabidopsis thaliana*, 7 in *Vitis vinifera*, 7 in *Oryza sativa*, 6 in *Solanum lycopersicum*, 11 in *Linum usitatissimum*, 21 in *Glycine max*, 3 in *Ricinus communis*, 6 in *Brachypodium distachyon*, 14 in *Zea mays*, 28 in *Brassica napus*, 5 in *Hordeum vulgare*, and 15 in *Pyrus bretschneideri* (Li et al., 2017a). In addition, 15 group IId members have been identified in *Gossypium hirsutum* (Dou et al., 2014). It has been shown that the group IId WRKY subfamily plays comprehensive roles in plant development and stress response, and transcriptomic and microarray data studies provide a great opportunity to study genes that might improve plant growth or stress tolerance (Ramamoorthy et al., 2008; Ding et al., 2015; Fan et al., 2015). In *Musa balbisiana*, group IId genes are abundant among the WRKY genes that regulate ethylene (ET)-related ripening, and 17 out of 25 group IId genes showed variable responses to ethylene (Goel et al., 2016). In *Oryza sativa*, 11 group IId WRKY genes showed variable responses in transcript abundance under abiotic (salt, drought, and cold) and various hormone treatments (Ramamoorthy et al., 2008). In *Gossypium hirsutum*, 9 group IId WRKY genes were differentially expressed at different stages of leaf senescence, 14 were differentially expressed in different tissues, and 6 were differentially expressed in different stresses (cold, salinity, ABA, drought, and alkalinity) (Dou et al., 2014). In *Hevea brasiliensis*, 8 group IId WRKY genes showed diverse transcript abundances in different tissues, and most genes were highly expressed in roots. In addition, *HbWRKY55*, -56, and -57 genes were induced differentially after exposure to jasmonic acid (JA) and ET treatments (Li et al., 2014).

In addition, functional verification of single group IId WRKY genes has been extensively performed in different species. In *Arabidopsis thaliana, Atwrky11* mutants revealed enhanced basal resistance, and *Atwrky11wrky17* double mutants worked partially redundantly with negative regulation (Journot-Catalino et al., 2006). In *Zea mays, ZmWRKY58* was induced by drought and salt stresses, and overexpression of *ZmWRKY58* enhanced drought and salt tolerance in transgenic rice plants by elevating their survival rates and relative water content (Cai et al., 2014b). With regard to *Triticum aestivum*, overexpression of its protein, *TaWRKY71-1*, in *Arabidopsis* resulted in a leaf hyponasty phenotype, followed by expression abundance changes in some leaf hyponasty-related genes (Qin et al., 2013). With regard to *Phyllostachys edulis*, overexpression of *PheWRKY72* in *Arabidopsis* conferred increased tolerance to drought stress at the seedling stage by regulating stoma closure (Li et al., 2017b). For *Gossypium hirsutum* proteins, ectopic expression of *GhWRKY15* in tobacco enhanced resistance to viral and fungal infections and improved the transcript levels of pathogen-related genes (Yu et al., 2012). Additionally, overexpression of *GarWRKY17* from *Gossypium aridum* in *Arabidopsis* positively regulated salt tolerance at both the germination and vegetative growth stages (Fan et al., 2015). Taken together, these findings demonstrated that group IId WRKY proteins are becoming more recognized as important regulators in multiple biological processes in plants.

Despite much progress in the identification of group IId WRKY proteins in plants, as far as we know, no detailed characterization has been focused on the evolution, expression profiles, and functional roles of the entire group IId WRKY subfamily in cotton. The released *Gossypium hirsutum* genome sequence and a publicly available database allow us to comprehensively characterize the group IId WRKY genes in this species. Furthermore, global analysis of the group IId WRKY genes will facilitate their functional study in cotton. Here, 34 group IId WRKY genes were identified in the upland cotton genome. Subsequently, we performed a comprehensive analysis of their chromosomal location, phylogeny, motif composition, gene structure, and Ka/Ks values. We further analyzed the expression patterns of the group IId WRKY genes in various tissues and in the presence of drought and salt stresses. Additionally, one of the group IId WRKY genes, Gh_A11G1801, was functionally characterized. In *Arabidopsis* plants, the Gh_A11G180 promoter conferred GUS expression was significantly induced by drought and JA treatments. The virus-induced gene silencing (VIGS) of Gh_A11G1801 in cotton plants could reduce drought tolerance. Our work provides basic information for further studies on the specific functions of these genes in cotton.

## Material and methods

### Identification and sequence analysis of putative group IId WRKY genes in cotton

Gu et al. performed a genome-wide identification of WRKY genes in cotton using *Gossypium hirsutum* sequences from the CottonGene database (http://www.cottongen.org) (Zhang et al., 2015; Gu et al., 2018b). Thirty-four group IId WRKY genes were identified through phylogenetic analysis (Gu et al., 2018b). In this study, the 34 group IId WRKY genes were used for further study. Molecular weights and isoelectric points were calculated using ExPASy (https://web.expasy.org/compute_pi/). A phylogenetic tree was constructed using MEGA 7 software. Exon-intron structures were constructed using the Gene Structure Display Server (GSDS) (http://gsds.cbi.pku.edu.cn/). Motifs were predicted online by MEME software (http://meme-suite.org/tools/meme). To determine the selection pressure, the Ka (non-synonymous substitution) and Ks (synonymous substitution) rates of duplicated genes were calculated on the PAL2NAL web server (http://www.bork.embl.de/pal2nal/#RunP2N).

### Expression profiles in different tissues and under various abiotic stresses

The ID numbers of the 34 group IId WRKY genes were submitted to CottonFGD (https://cottonfgd.org/analyze/) to obtain gene expression levels (Zhu et al., 2017). The data were from different tissues: root, stem, leaf, calycle, torus, petal, stamen, pistil, fiber at 10 days post-anthesis, and ovule at 10 days post-anthesis. For abiotic stresses, the data (at 1, 3, 6, and 12 h) were from samples that had received polyethylene glycol (PEG) and salt treatments. The expression levels of genes were indicated using the values of the fragments per kilobase of exon per million reads mapped (FPKM).

### Abiotic stress treatments in cotton seedlings

Seeds of cotton variety CCRI10 were planted in pots in a greenhouse at 25°C with a 16-h light/8-h dark cycle. Ten days later, the uniform seedlings were subjected to drought and salt treatments. For drought treatment, a strong water absorbent PEG6000 that induces osmotic stress was used to mimic the water deficit or drought. In addition, NaCl was used for salinity stress. For both treatments, the seedlings were irrigated with 15% PEG6000 (w/v) and 200 mM NaCl. Cotyledon samples were collected at 0, 2, 4, 6, 8, and 12 h. Three independent biological repetitions were used. The samples were quickly frozen in liquid nitrogen for RNA extraction.

### RNA extraction and quantitative RT-PCR (qRT-PCR)

Total RNA was extracted from the samples using the RNAprep PurePlant Kit (Tiangen, China). cDNA was synthesized using a PrimeScript™ RT reagent kit with gDNA Eraser (TaKaRa, Dalian, China). qRT-PCR was carried out using GoTaq® qPCR Master Mix (Promega) in a 7500 Real-Time PCR system (Applied Biosystems) with the following reaction mix: 10 μl of GoTaq® qPCR Master Mix (2×), 0.4 μl of upstream primer (10 μM), 0.4 μl of downstream primer (10 μM), 2 μl of cDNA, and 7.2 μl of ddH_2_O. The amplifications were performed using the following protocol: a pre-denaturation step at 95°C for 2 min, followed by 40 cycles of 95°C for 3 s and 60°C for 30 s. *GhActin* and *AtActin2* were used as reference genes. Three technical repetitions were performed for each sample. The primers designed by Oligo 7 are listed in Supplementary Table S1.

### Stress treatment and β-glucuronidase (GUS) staining of transgenic *Arabidopsis* with the Gh_A11G1801 promoter

The Gh_A11G1801 gene is corresponding to *GhWRKY42* according to Dou et al. (2014), and transgenic plants of its promoter, *Gh_A11G1801p::GUS* (*ProGhWRKY42*::GUS), were obtained previously (Gu et al., 2018a). To investigate promoter-induced GUS expression under stress conditions, transgenic *Arabidopsis* plants with foreign promoters were used for different treatments. The *Arabidopsis* seeds were germinated on 1/2 MS solid medium, and ten-day-old seedlings were transferred to 1/2 MS liquid medium supplemented with 200 mM D-mannitol, 150 mM NaCl, 100 μM abscisic acid (ABA), 500 μM salicylic acid (SA), or 100 μM JA incubating for 3 h. Plants immersed in 1/2 MS liquid medium were used as a control (CK). After treatment, the *Arabidopsis* plants were assayed for GUS staining using the GUS Staining Kit (RealTimes, Beijing). After staining, the samples were decolored 3–5 times in 75% alcohol until positive blue spots were stable. For further analysis, at least 30 seedlings from each treatment were collected to identify the transcript levels of the GUS gene. The experiments were performed with three independent biological repetitions.

### VIGS and drought treatment

A 404-bp fragment of Gh_A11G1801 was amplified and inserted into the XbaI and BamHI sites of the pYL156 vector. The recombinantly constructed vector was transformed into *Agrobacterium tumefaciens* GV3101. GV3101 strains carrying pYL156 (empty vector), pYL156-Gh_A11G1801 (VIGS), pYL156-CLA1 (positive control), and pYL192 (helper vector) were shaken in LB liquid medium containing 50 μg/mL kanamycin, rifampicin and gentamycin using a shaker at 28°C with 180 rpm. When the OD600 value of the culture reached 1.5–2.0, the culture was centrifuged, re-suspended, and adjusted to OD600 = 1.5 with infiltration buffer (10 mM MgCl_2_, 10 mM MES, and 200 μM acetosyringone). The suspension was left at room temperature for 3 h. Before injection, we mixed the suspensions of pYL156, pYL156-Gh_A11G1801, and pYL156-*GrCL1* with pYL192 in a 1:1 ratio. Each mixture was injected into the underside of cotyledons of cotton variety CCRI10 plants. The injected plants were left in dark overnight and transferred to a greenhouse at 25°C with a 12-h light/12-h dark cycle. The plants injected with pYL156 empty control and pYL156-Gh_A11G1801 were subjected to drought treatment after four weeks. For drought treatments, two methods were applied. The pYL156 and pYL156-Gh_A11G1801 plants were placed on the same plates and irrigated with 15% PEG6000 (w/v) or were not provided water to detect their drought response. The experiments were performed with three independent biological repetitions.

### Measurement of malondialdehyde (MDA) and catalase (CAT) content

The MDA content was determined using a Malondialdehyde (MDA) Assay Kit (Solarbio, Beijing, China). The CAT content was determined using a Micro Catalase (CAT) Assay Kit (Solarbio, Beijing, China). The experimental procedure was carried out according to the instructions. During measurement, ~0.1 g leaf samples of pYL156 and pYL156-Gh_A11G1801 plants under control and water-withholding treatments were used for content determination using a spectrophotometer. The experiments were performed with three independent biological repetitions.

## Results

### Characterization of group IId WRKY genes in cotton

We identified a total of 34 distinct group IId WRKY genes in the whole upland cotton genome (Table 1). As shown in Table 1, 14 genes were located on the A-subgenome, 17 genes on the D-subgenome, and 3 genes on the scaffold. These candidate genes had genomic DNA sequences ranging from 768 to 11276 bp in length, coding sequences ranging from 243 to 1077 bp and encoded proteins ranging from 80 to 358 amino acids. The GC content of the coding sequences ranged from 37.5 to 51.2%. The molecular weights of these proteins ranged from 9.089 to 39.689 kDa, and their isoelectric points ranged from 4.887 to 10.526. However, most genes (29 out of 34) contained three exons, 3 genes (Gh_A12G1124, Gh_D06G0174 and Gh_Sca005611G02) contained 2 exons, and 2 genes (Gh_A08G1914 and Gh_D08G2032) contained 4 exons.

**Table 1:** Basic information on 34 group IId WRKY genes in cotton.

**Gene ID**	**Chromosome**	**Gene Start**	**Gene End**	**Gene Length (bp)**	**CDS Length (bp)**	**Protein Length (aa)**	**CDS GC Content (%)**	**Molecular Weight (kDa)**	**Isoelectric Point**	**Exon Number**
Gh_A02G1488	A02	81025465	81026529	1065	876	291	41.1	32.216	10.052	3
Gh_A05G0528	A05	5619737	5621279	1543	993	330	50.1	36.445	9.595	3
Gh_A05G1774	A05	18705599	18707150	1552	1077	358	44.2	39.346	10.087	3
Gh_A05G2237	A05	26044000	26045437	1438	1065	354	42.8	38.706	10.247	3
Gh_A06G0179	A06	2021580	2023105	1526	957	318	41.5	34.9	10.016	3
Gh_A06G2118	scaffold1602_A06	13527	15406	1880	1053	350	49.9	37.87	10.348	3
Gh_A07G0017	A07	160547	161753	1207	1008	335	51.2	36.134	10.011	3
Gh_A07G1912	A07	74734277	74735779	1503	996	331	44.1	36.938	9.948	3
Gh_A08G1540	A08	92668103	92670237	2135	879	292	41.8	32.662	9.999	3
Gh_A08G1914	A08	99868631	99870753	2123	1044	347	37.5	39.689	9.959	4
Gh_A11G0997	A11	10941331	10942459	1129	747	248	42.5	27.797	4.887	3
Gh_A11G1172	A11	14300105	14301287	1183	1011	336	44.3	37.322	10.057	3
Gh_A11G1801	A11	34535928	34537120	1193	1038	345	46.3	37.86	9.697	3
Gh_A11G2258	A11	77607472	77608657	1186	981	326	39.8	36.81	10.422	3
Gh_A12G1124	A12	64643993	64644854	862	798	265	39.7	30.085	6.434	2
Gh_D03G0226	D03	2379359	2380514	1156	951	316	40.6	35.031	10.018	3
Gh_D05G0648	D05	5194550	5196097	1548	993	330	49.9	36.58	9.624	3
Gh_D05G1968	D05	18136315	18137847	1533	1077	358	43.9	39.351	10.099	3
Gh_D05G2499	D05	25134730	25136167	1438	1065	354	43	38.736	10.306	3
Gh_D06G0174	D06	1745417	1746184	768	243	80	41.2	9.089	8.246	2
Gh_D06G0175	D06	1746646	1748213	1568	957	318	41.4	34.918	9.927	3
Gh_D06G1121	D06	25910270	25911911	1642	1053	350	51	37.798	10.22	3
Gh_D07G0023	D07	246278	247486	1209	1008	335	51.2	36.298	9.969	3
Gh_D07G2135	D07	51630068	51631580	1513	996	331	44.2	36.946	9.909	3
Gh_D08G1841	D08	55326801	55328428	1628	879	292	41.1	32.812	10.106	3
Gh_D08G2032	D08	58840331	58851606	11276	963	320	42.9	35.883	10.526	4
Gh_D08G2279	D08	62352024	62353273	1250	1017	338	37.7	38.514	9.992	3
Gh_D11G1141	D11	10515259	10516428	1170	807	268	43.1	30.016	4.908	3
Gh_D11G1328	D11	12797394	12798583	1190	1011	336	44.3	37.223	9.973	3
Gh_D11G1963	D11	25270922	25272114	1193	1038	345	46.5	37.862	9.59	3
Gh_D11G2566	D11	53173124	53174303	1180	975	324	39.8	36.608	10.509	3
Gh_D12G1253	D12	40607658	40608518	861	438	145	41.8	16.281	5.341	3
Gh_Sca005611G01	scaffold5611	316	2037	1722	966	321	43.2	35.973	10.413	3
Gh_Sca005611G02	scaffold5611	10059	11812	1754	1053	350	42.3	39.658	10.352	2

### Chromosomal location of group IId WRKY genes

**Figure 1:**
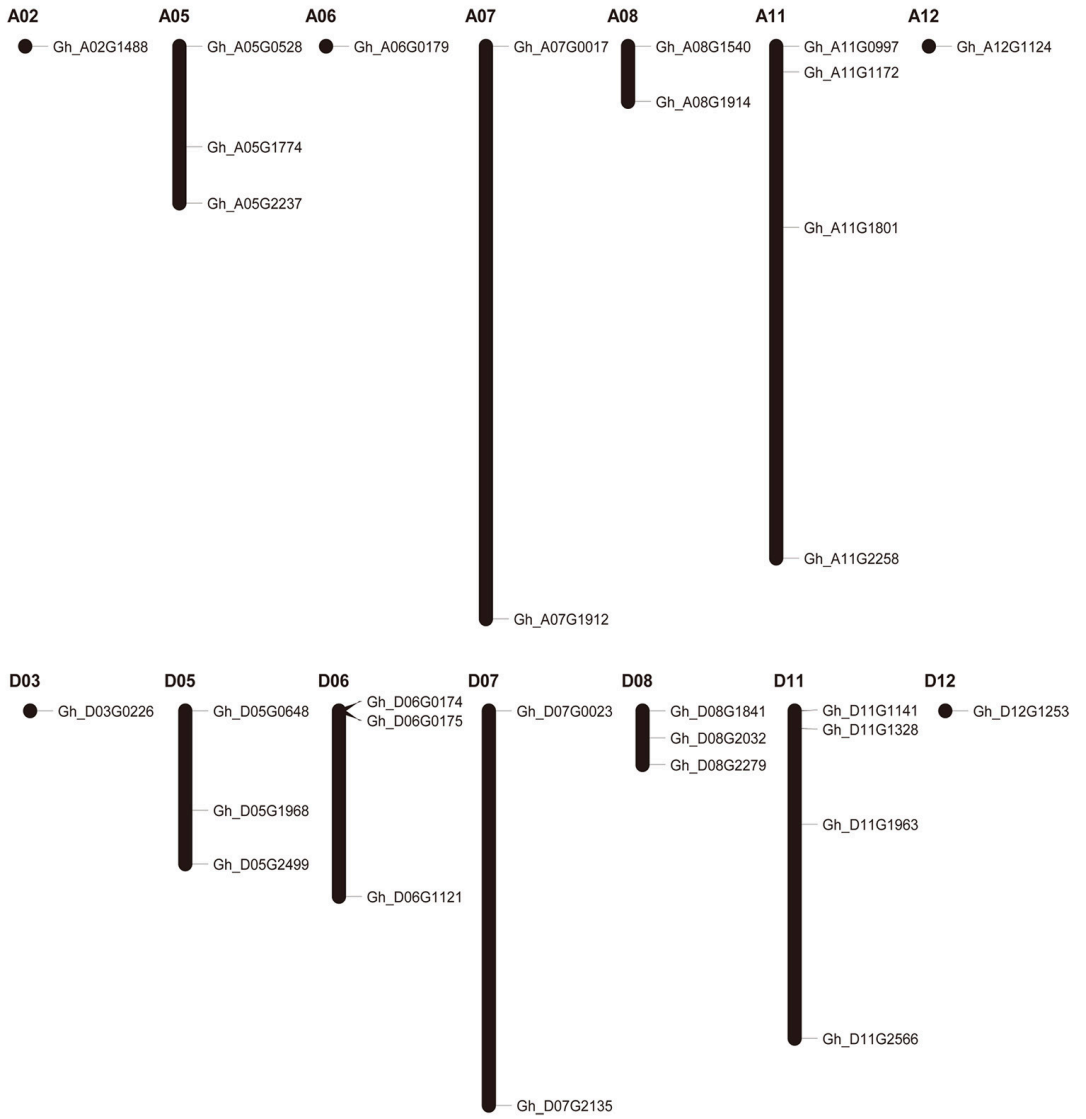
Chromosomal locations of group IId WRKY genes in the *Gossypium hirsutum* genome.

To study the chromosomal distribution of group IId WRKY genes, we drew the physical location of these genes on cotton chromosomes. Thirty-one candidate group IId WRKY genes were mapped to 14 of the total 24 chromosomes with a non-random distribution; three genes, Gh_A06G2118, Gh_Sca005611G01 and Gh_Sca005611G02, are not located on any one chromosome because they were located on scaffolds. The mapped chromosomes were A02/05/06/07/08/11/12 and D03/05/06/07/08/11/12, while chromosomes A01/03/04/09/10 and D01/02/04/09/10 contained no genes. The number of genes on mapped chromosomes varied from 1 to 4. Among these chromosomes, 5 chromosomes contained only 1 gene, 3 contained 2 genes, 4 contained 3 genes, and 2 contained 4 genes (Figure 1).

### Phylogenetic and motif analysis of group IId WRKY genes in different species

**Figure 2:**
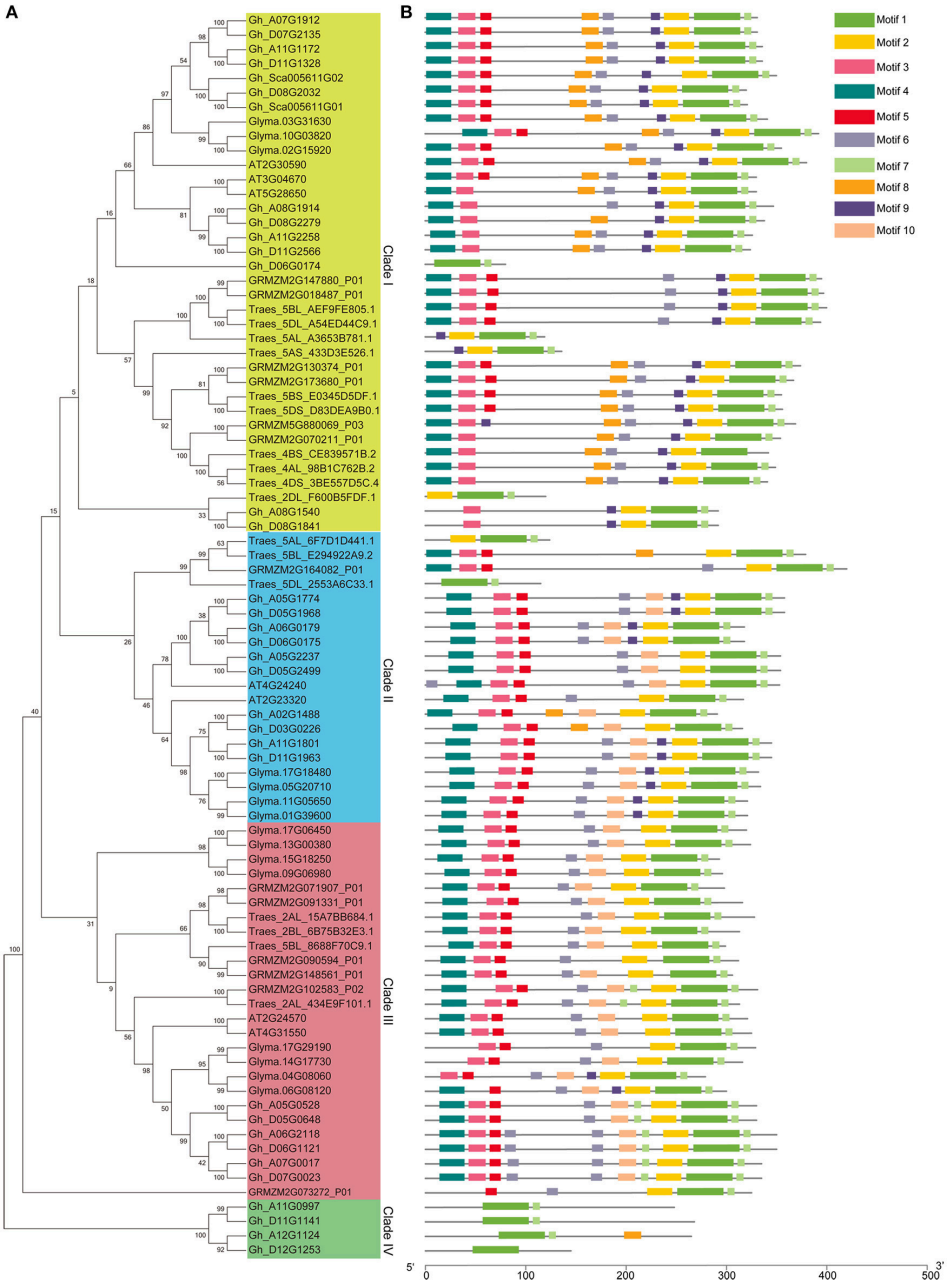
Phylogenetic tree and motif composition of group IId WRKY members from different species. **(A)** Phylogenetic tree of group IId WRKY members from different species. A phylogenetic tree was constructed by using MEGA 7 software with the neighbor-joining method. **(B)** Motif composition of group IId WRKY members from different species. The motifs were predicted by MEME software.

The phylogenetic tree was constructed to investigate the relationship among the group IId WRKY genes using the 86 protein sequences from different species, including cotton, wheat, rice, soybean, and *Arabidopsis* (Figure 2A). Through the evolution analysis, a reasonable rule was observed among these genes. The phylogenetic tree was mainly classified into four branches (Clades I–IV) (Figure 2A). Clade IV has the fewest group IId members (4), while Clade I has the most members (36), followed by Clade III (26), and Clade II (20). Clades I, II, and III contain at least one group IId WRKY member from each species, whereas Clade IV contains only cotton genes (Figure 2A). Based on the phylogenetic tree, two genes were clustered together to form many gene pairs (Figure 2A). A total of 36 gene pairs were formed, 16 of which were from cotton. In each cotton gene pair, one gene was from the A genome and the other was from the D genome. In addition, both genes in each pair except one pair (Gh_A02G1488/Gh_D03G0226) were located on the same numbered chromosome (Figures 1, 2A).

All group IId WRKY proteins were submitted to MEME software for the prediction of their conserved motifs. A total of 10 distinct motifs were obtained, and proteins with the same motif composition were preferentially clustered together (Figure 2B). As shown in Figure 2B, most group IId WRKY members in the same branch, especially those with the closest relationship, generally have a common motif pattern. In addition, both genes in each gene pair possessed the same motif composition, implying their functional similarity at the protein level (Figure 2B). Moreover, Clade I and Clade II mainly contained nine motifs, although Motif 10 was not present in Clade I and Motif 2 was not present in Clade II. Clade III mainly contained eight motifs but not Motifs 8 and 9. However, Clade IV contained the minimum number of motifs (1–3) (Figure 2B), probably due to pseudogenes, sequencing errors or incomplete assemblies (Rinerson et al., 2015).

### Phylogenetic and gene structure analysis of group IId WRKY genes in cotton

**Figure 3:**
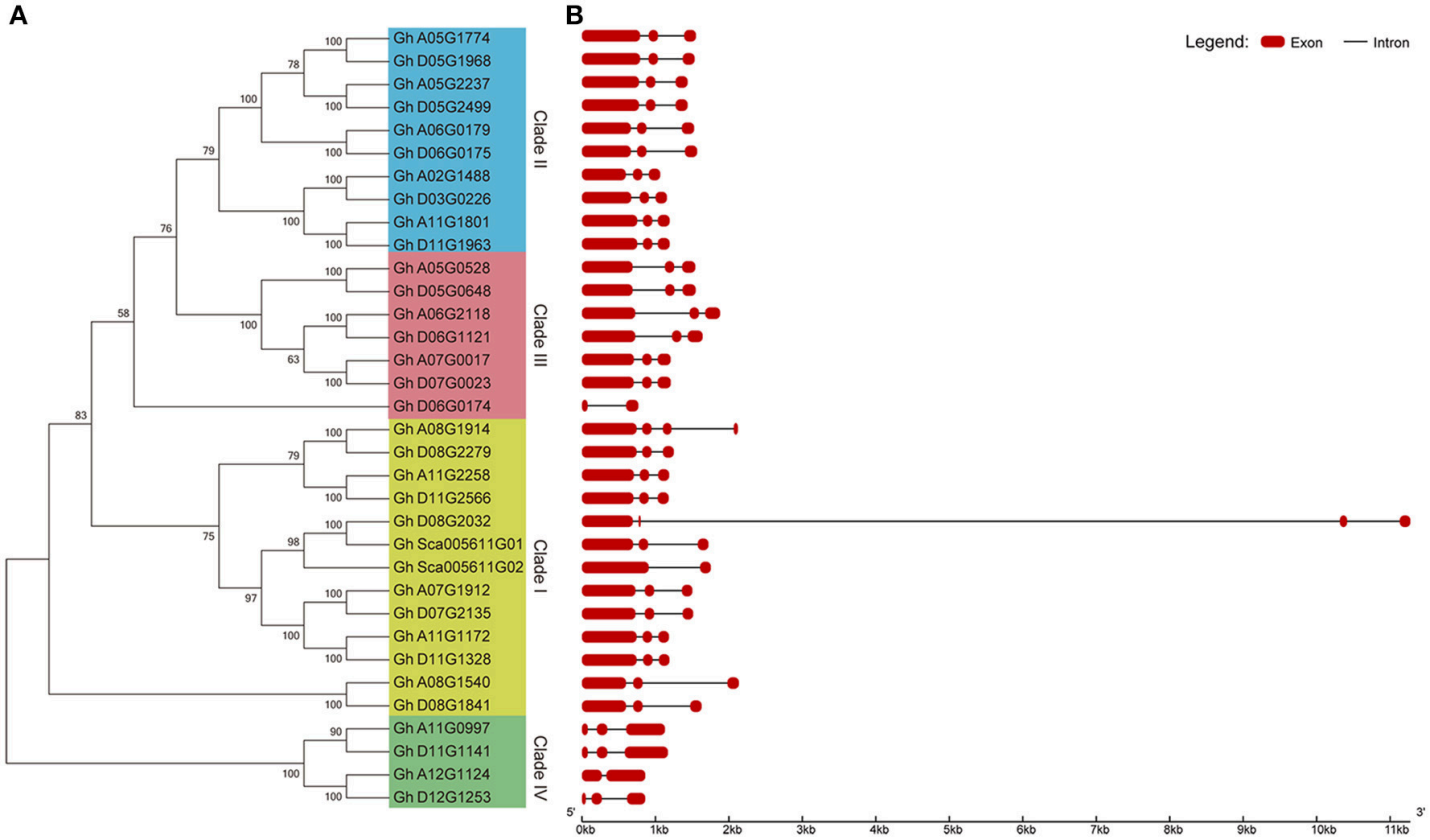
Phylogenetic tree and exon-intron structure analysis of group IId WRKY genes in cotton. **(A)** Phylogenetic analysis of 34 group IId WRKY genes. A phylogenetic tree was constructed by using MEGA 7 software with the neighbor-joining method. **(B)** Exon-intron structure of 34 group IId WRKY genes. The exon-intron structure was generated on GSDS software online. Red indicates exons, and black indicates introns.

To further analyse the gene pairs among group IId WRKY genes in cotton, a phylogenetic tree and exon-intron structure were constructed using all 34 group IId *GhWRKY* protein sequences. Similar to Figure 2A, the 34 genes were classified into four groups (Clade I-IV), and 32 genes clustered together in 16 gene pairs (Figure 3A). To explore the structural differences in each pair, the exon-intron structure was determined. As in Table 1, most group IId WRKY genes generally contained 3 exons and two introns (Figure 3B). In addition, both genes in most pairs possessed similar exon-intron structure and gene length except three pairs (Gh_A08G1914/Gh_D08G2279, Gh_D08G2032/Gh_Sca005611G01, and Gh_A08G1540/Gh_D08G1841) have different lengths of introns or different numbers of exons (Figure 3B).

### Ka/Ks analysis

An alignment sequence covering more than 80% of the longest gene and a sequence similarity exceeding 70% were used as standards for inferring gene duplication events (Gu et al., 2002). Based on these criteria for gene duplication, 15 gene pairs participated in gene duplication events, and their shared protein identity varies from 89.87 to 98.74% (Table 2). The Ka/Ks ratios for duplicated group IId WRKY genes were determined. The Ka/Ks ratio of Gh_A11G2258/Gh_D11G2566 was >1. However, the Ka/Ks ratios of the remaining 14 gene pairs were < 1, indicating that they have undergone purifying selection (Table 2).

**Table 2:** The Ka/Ks ratios for duplicated group IId WRKY genes.

**Paralogues**	**Protein identities (%)**	**Ka**	**Ks**	**Ka/Ks**	**Purifying selection**
Gh_A02G1488/Gh_D03G0226	89.87	0.0128	0.0274	0.4656	YES
Gh_A05G0528/Gh_D05G0648	96.06	0.0197	0.0395	0.4992	YES
Gh_A05G1774/Gh_D05G1968	97.99	0.0084	0.0466	0.1793	YES
Gh_A05G2237/Gh_D05G2499	98.59	0.0065	0.0075	0.8587	YES
Gh_A06G0179/Gh_D06G0175	98.74	0.0054	0.0435	0.1232	YES
Gh_A06G2118/Gh_D06G1121	96.86	0.0151	0.0516	0.2931	YES
Gh_A07G0017/Gh_D07G0023	97.02	0.0117	0.0582	0.2005	YES
Gh_A07G1912/Gh_D07G2135	96.98	0.0130	0.0399	0.3248	YES
Gh_A08G1540/Gh_D08G1841	96.92	0.0151	0.0565	0.2680	YES
Gh_A08G1914/Gh_D08G2279	91.93	0.0325	0.0563	0.5775	YES
Gh_A11G0997/Gh_D11G1141	91.42	0.0052	0.0190	0.2737	YES
Gh_A11G1172/Gh_D11G1328	98.51	0.0074	0.0279	0.2663	YES
Gh_A11G1801/Gh_D11G1963	98.26	0.0081	0.0453	0.1779	YES
Gh_A11G2258/Gh_D11G2566	97.24	0.0096	0.0089	1.0774	NO
Gh_D08G2032/Gh_Sca005611G01	96.57	0.0139	0.0555	0.2504	YES

### Expression profiles of group IId WRKY genes in different tissues

**Figure 4:**
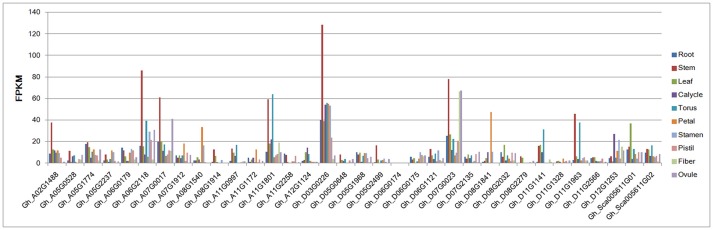
Expression patterns of group IId WRKY genes in different tissues from RNA-seq data. The scale represents the FPKM values. The tissues were from root, stem, leaf, calycle, torus, petal, stamen, pistil, fiber at 10 days post-anthesis and ovule at 10 days post-anthesis.

To clarify the potential functional roles of the group IId WRKY genes in cotton, the publicly available RNA-seq data were used to identify the expression pattern of group IId WRKY genes in different tissues. Ten tissue organs, including root, stem, leaf, calycle, torus, petal, stamen, pistil, fiber at 10 days post-anthesis and ovule at 10 days post-anthesis, were used in the expression detection. As shown in Figure 4, no transcript expression was identified for the Gh_A05G1774 gene, whereas the remaining 33 genes exhibited distinctive expression patterns in different tissues. Generally, 22 of the 34 genes showed relatively low expression levels, with FPKM < 20 in all tissues. The remaining 12 genes had relatively high expression levels and were also highly expressed in specific tissues with FPKM>20 or even >120. For example, Gh_A02G1488, Gh_A08G1540, Gh_D08G1841, Gh_D11G1141, Gh_D12G1253, and Gh_Sca005611G01were highly specifically expressed in stem (FPKM>20), petal (FPKM>20), petal (FPKM>40), torus (FPKM>20), calycle (FPKM>20), and leaf (FPKM>20), respectively. Additionally, Gh_A06G2118, Gh_A07G0017, Gh_A11G1801, Gh_D03G0226, Gh_D07G0023, Gh_D11G1963 were highly expressed in stem tissue, with FPKM>40 (Figure 4).

### Expression profiles of group IId WRKY genes under drought and salt treatments

**Figure 5:**
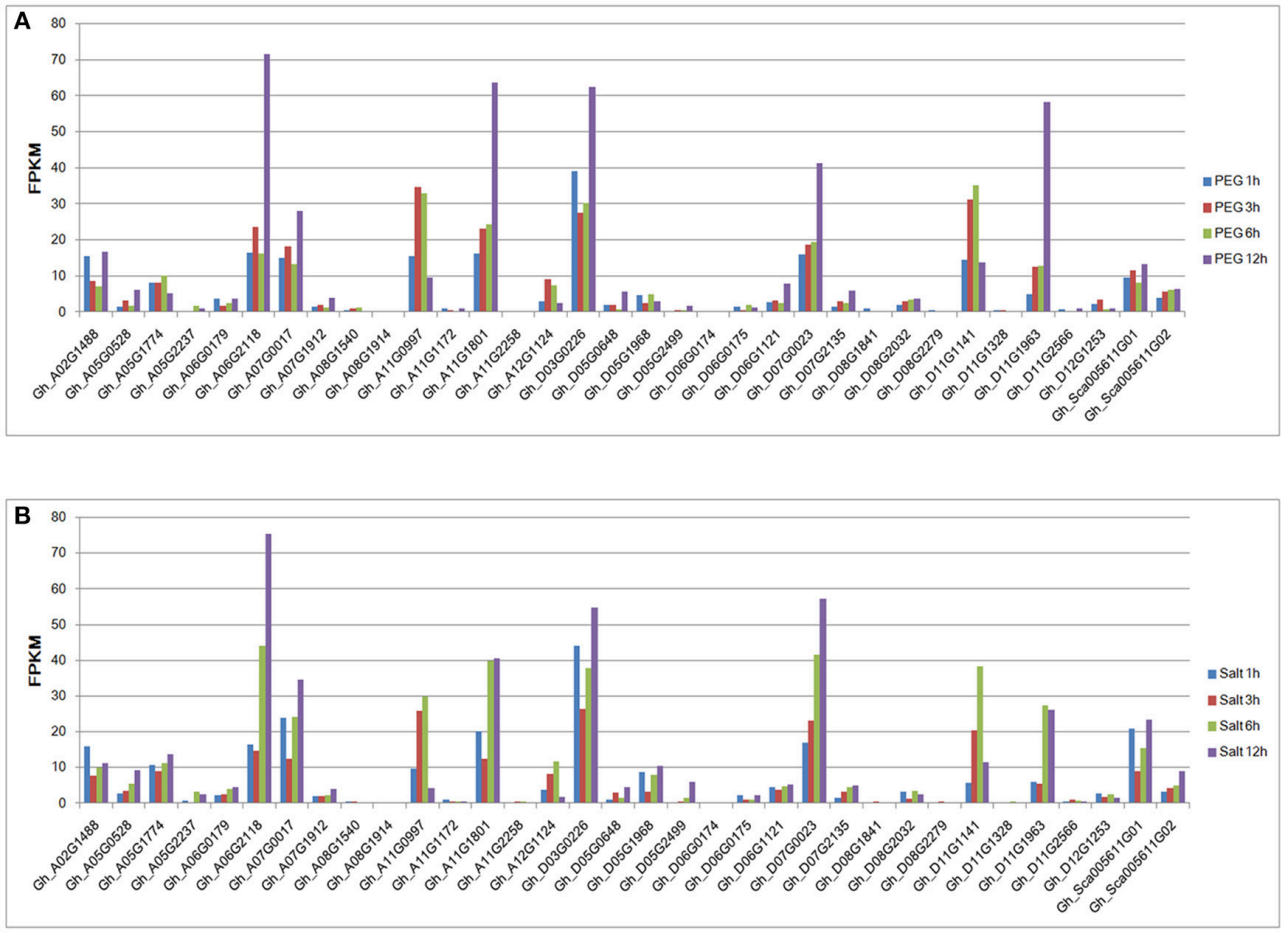
Expression patterns of group IId WRKY genes under drought and salt stresses from RNA-seq data. **(A)** The expression patterns of group IId WRKY genes under drought stress. **(B)** The expression patterns of group IId WRKY genes under salt stress. The scale represents the FPKM values.

The publicly available RNA-seq data were also used to analyze the gene expression patterns under drought and salt conditions. The expression levels of most group IId WRKY genes were induced by abiotic stresses. However, the transcript abundance of the Gh_A05G1774 gene was not detected in the transcriptome database (Figure 5). Among the 33 remaining expressed genes, 10 were highly expressed (FPKM>10), while the other 23 genes had relatively low expression levels (FPKM < 10) under drought treatment in time series (Figure 5A). Under salt treatment, 12 genes had high expression levels, and their FPKM values were >10, while the other 21 genes had relatively low expression levels (FPKM < 10) in the time series (Figure 5B). Under both stress conditions, most genes with high expression levels (FPKM>10) had their levels peak at 12 h; Gh_A11G0997 (3 h) and Gh_D11G1141 (6 h) in the drought treatment and Gh_A02G1488 (1 h), Gh_A11G0997 (6 h), Gh_D11G1141 (6 h), and Gh_D11G1963 (6 h) in the salt treatment were exceptions (Figure 5).

### qRT-PCR analysis of the candidate group IId WRKY genes in response to drought and salt treatments

**Figure 6:**
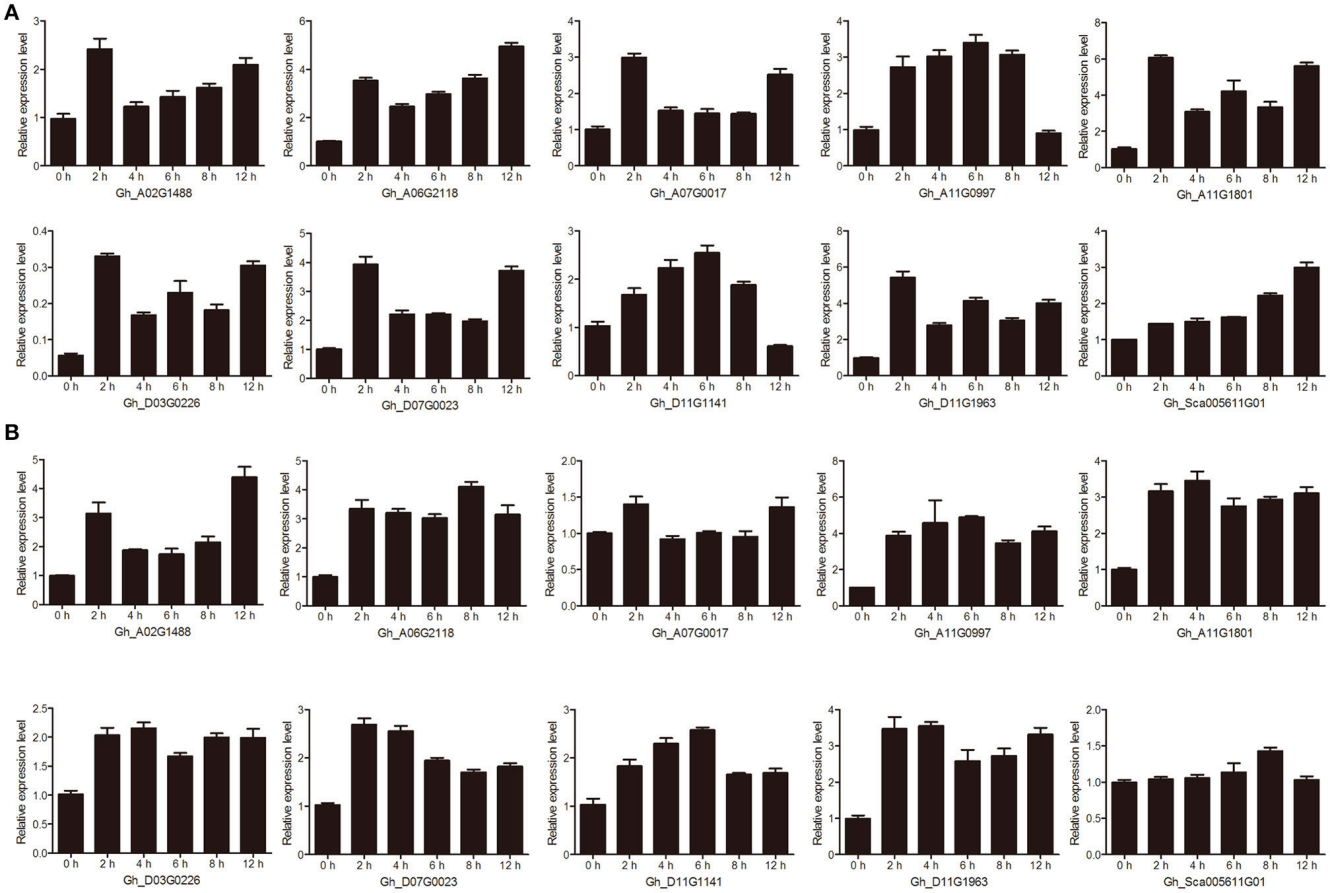
The relative transcript abundances of ten candidate group IId WRKY genes under drought and salt treatments were examined by qRT-PCR. **(A)** qRT-PCR analysis of ten candidate group IId WRKY genes under drought treatment. **(B)** qRT-PCR analysis of ten candidate group IId WRKY genes under salt treatment. Ten-day-old cotton seedlings were subjected to either 15% PEG6000 or 200 mM NaCl. The cotyledon samples were collected at 0, 2, 4, 6, 8, and 12 h. Three biological repeats and three technical repetitions were performed. *GhActin* was used as an internal reference. The data are presented as the means ± standard errors (SEs) from three biological replicates.

We performed drought and salt treatments on cotton seedlings to further examine the stress effect on the transcript abundance of genes with qRT-PCR. Ten group IId WRKY genes (Gh_A02G1488, Gh_A06G2118, Gh_A07G0017, Gh_A11G0997, Gh_A11G1801, Gh_D03G0226, Gh_D07G0023, Gh_D11G1141, Gh_D11G1963, and Gh_Sca005611G01) that were reported in the transcriptome database to be highly expressed were used to assess responses to drought and salt treatments. All of these genes were quickly induced at 2 h and differentially upregulated at different time points (Figure 6).

Under drought treatment, some genes displayed basically similar expression patterns (Figure 6A). For example, Gh_A02G1488, Gh_A06G2118, Gh_A07G0017, Gh_A11G1801, Gh_D03G0226, Gh_D07G0023, and Gh_D11G196 were strongly elevated at 2 h, decreased in the subsequent 4–8 h and increased again by 12 h (Figure 6A). Nevertheless, the expression of Gh_A11G0997 and Gh_D11G1141 increased gradually, reaching maximum transcript levels at 8 h, then decreased gradually and finally maintained minimum levels at 12 h (Figure 6A). However, the mRNA levels of Gh_Sca005611G01 showed a gradual rising trend and reached their highest level at 12 h (Figure 6A). Under salt treatment, Gh_A02G1488 and Gh_A07G0017 genes were induced at 2 h, and after a low expression level period (4–8 h), the expression level increased again at 12 h. Generally, the expression levels of other genes showed a trend of increasing first and then decreasing. However, the Gh_Sca005611G01 gene was weakly induced at only 8 h (Figure 6B).

### Gh_A11G1801 promoter activity in transgenic arabidopsis plants under different treatments

**Figure 7:**
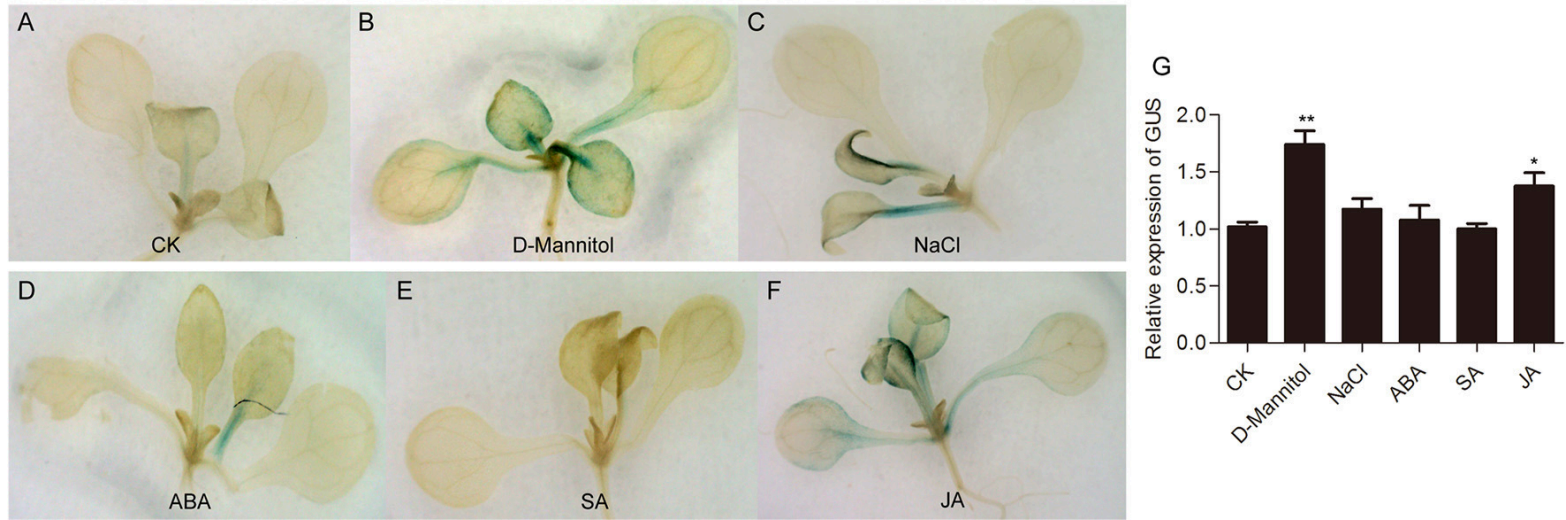
GUS activity analysis of *Gh_A11G1801p::GUS* transgenic *Arabidopsis* under various stresses. GUS staining of *Gh_A11G1801p::GUS* transgenic *Arabidopsis* under **(A)** CK, **(B)** 200 mM D-mannitol, **(C)** 150 mM NaCl, **(D)** 100 μM ABA, **(E)** 500 μM SA, and **(F)** 100 μM JA treatments. The 10-day-old seedlings grown on 1/2 MS solid medium were transferred to 1/2 MS liquid medium supplemented with 200 mM D-mannitol, 150 mM NaCl, 100 μM ABA, 500 μM SA, or 100 μM JA. After 3 h of treatment, the seedling samples were used for GUS staining. *Arabidopsis* seedlings that had been placed in 1/2 MS liquid medium without any stress treatment were used as controls. **(G)** The transcript levels of the GUS gene in *Gh_A11G1801p::GUS* transgenic *Arabidopsis* under various treatments. *AtActin2* was used as the reference control. The data are presented as the means ± SEs from three biological replicates. ** and * indicate statistical significance at the 0.01 and 0.05 probability levels, respectively.

To evaluate the stress response of the Gh_A11G1801 promoter, GUS activity in *Gh_A11G1801p::GUS* transgenic plants was assayed under drought, salt, ABA, SA, and methyl jasmonate (JA) treatments. The CK plants revealed weak GUS activity in petiole and true leaves (Figure 7A). Meanwhile, weak GUS staining was also observed in the seedlings treated with 150 mM NaCl (Figure 7C), 100 μM ABA (Figure 7D), or 500 μM SA (Figure 7E). However, GUS activity was strongly induced in the petiole, true leaves and cotyledons after either 200 mM D-mannitol (Figure 7B) or 100 μM JA (Figure 7F) treatments. In addition, the GUS activity was further determined by the transcript levels of the GUS gene. It was found that the expression of the GUS gene significantly increased under D-mannitol and JA treatments compared with CK (Figure 7G). However, we found no significant difference in GUS expression between CK and NaCl, ABA or SA treatments (Figure 7G).

### Silencing Gh_A11G1801 reduced tolerance to drought stress in cotton

**Figure 8:**
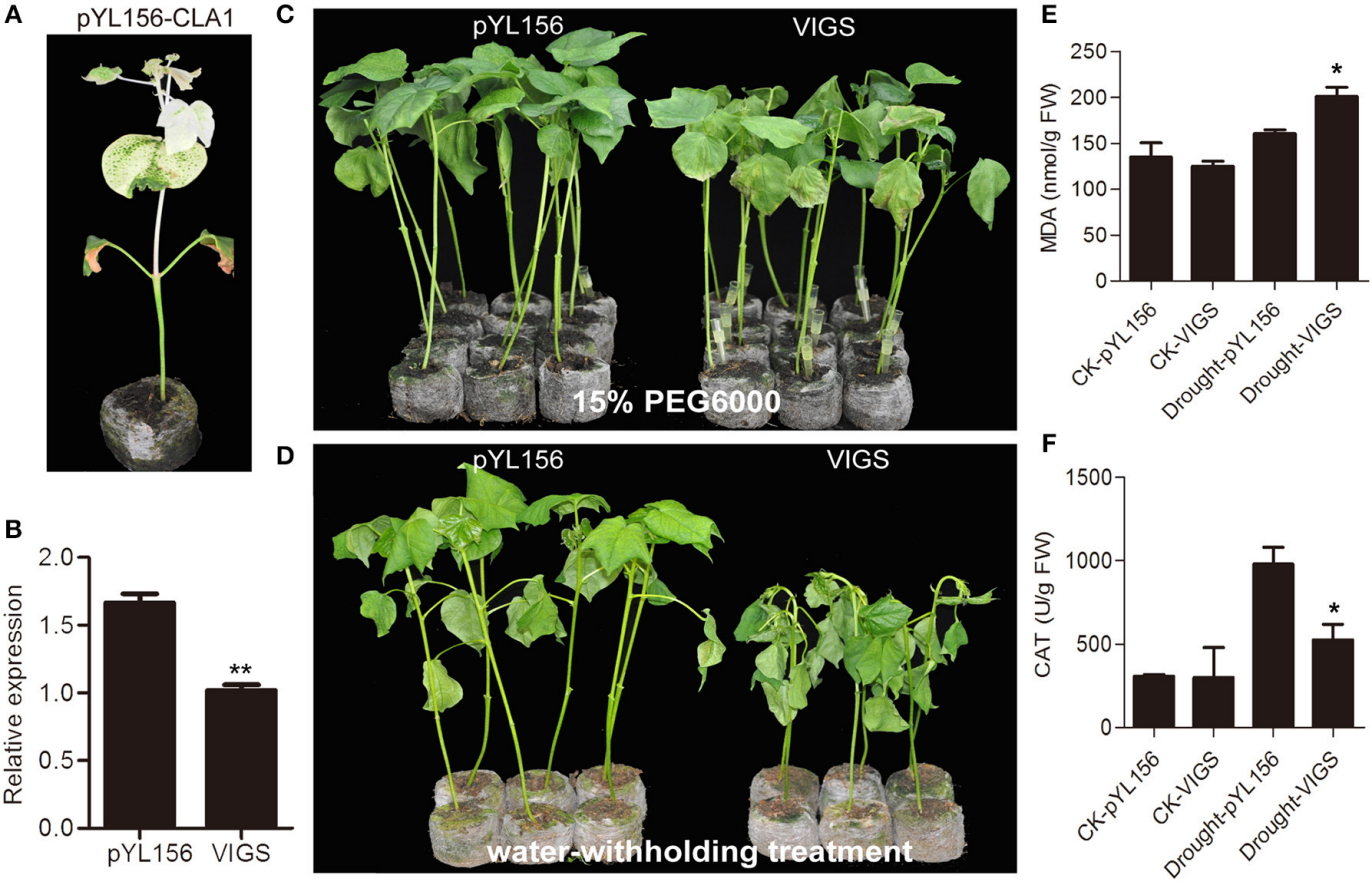
Silencing Gh_A11G1801 via VIGS increased sensitivity to drought stress in cotton. **(A)** Plant phenotypes of positive control plants. **(B)** Expression level of Gh_A11G1801 in empty control and VIGS plants. **(C)** Phenotype of empty control and VIGS plants under 15% PEG6000 treatment. After three weeks of injection, the VIGS plants were treated with 15% PEG6000 for 8 days. **(D)** Phenotype of empty control and VIGS plants under water deficiency. After three weeks of injection, the VIGS plants were treated with water shortage for 7 days. **(E)** The MDA content of empty control and VIGS plants under normal growth and water-withholding conditions. **(F)** The CAT content of empty control and VIGS plants under normal growth and water-withholding conditions. CK-pYL156 and CK-VIGS indicate plants under normal growth conditions. Drought-pYL156 and Drought-VIGS indicate plants under water-withholding conditions. *GhActin* was used as the reference control. The bars represent the means ± SEs from three independent experiments. ** and * indicate statistical significance at the 0.01 and 0.05 probability levels, respectively.

To investigate the role of Gh_A11G1801 in the response to drought treatment, we performed a VIGS assay to decrease the expression of the endogenous Gh_A11G1801 gene in cotton. The appearance of an albino phenotype on pYL156-CLA1 cotton plants ensured the success of the VIGS experiment (Figure 8A). The expression levels of Gh_A11G1801 in pYL156-Gh_A11G1801 (VIGS) and pYL156 (empty vector) cotton plants were determined via qRT-PCR. As shown in Figure 8B, the drastically reduced expression of the Gh_A11G1801 gene in the VIGS plants demonstrated that the gene had been successfully knocked down. The VIGS and empty control plants were subjected to drought stress. The plants were irrigated with 15% PEG6000, and after 8 days of treatment, the VIGS plants showed a more severe wilting phenotype on leaves than the control plants (Figure 8C). In addition, the plants were used for a water-withholding treatment. Seven days later, similar to the PEG6000 treatment, the VIGS plants presented obvious wilting, while the controls were less influenced (Figure 8D). Additionally, the MDA and CAT contents were examined to explore the potential mechanisms underlying the reduced drought tolerance in VIGS plants. There was no difference in the MDA and CAT contents under normal growth conditions and an increasing accumulation of the MDA and CAT contents under water-withholding conditions (Figures 8E,F). Under water-withholding conditions, more MDA content was observed in the VIGS plants than in the pYL156 control plants (Figure 8E), whereas less CAT content was observed in the VIGS plants than in the pYL156 control plants (Figure 8F). The results demonstrated that silencing of the Gh_A11G1801 gene reduced drought tolerance in cotton.

## Discussion

Cotton is an important economic crop that provides large amounts of fiber, oil, and biofuel products. Despite its importance, our knowledge about gene functions in cotton is still very limited. The group IId WRKY genes are a subfamily of WRKY TFs with broad roles in biological processes, particularly in plant responses to abiotic stresses (Chen et al., 2012). Systematic and comprehensive analyses of the WRKY gene family have been reported in various species, and these analyses provide important insights into understanding the functions of the group IId WRKY genes in cotton. The release of the *Gossypium hirsutum* genomic sequence provides a good resource for the comprehensive analysis of the group IId WRKY genes in cotton (Zhang et al., 2015).

In this study, we obtained a total of 34 group IId WRKY genes in cotton. This number of group IId WRKY gene members is larger than the number found in 15 other plant species (Li et al., 2017a), which is likely due to the polyploidy of cotton (Otto, 2007; Zhang et al., 2015; Li et al., 2017a). The group IId members account for 9.6% (34/239) of all *GhWRKY* genes (Gu et al., 2018b), which is similar to the ratio of 9.7% (7/72) in *Arabidopsi*s (Eulgem et al., 2000) and 10.3% (6/58) in physic nut (Xiong et al., 2013). Studies on the group IId WRKY subfamily in cotton have also been reported in other literature. Cai et al. identified 15 group IId WRKY members in *Gossypium raimondii* (Cai et al., 2014a), Fan et al. identified 15 in *Gossypium aridum* (Fan et al., 2015), and Dou et al. identified 15 in *Gossypium hirsutum* (Dou et al., 2014). We have clearly identified more group IId WRKY genes than other reports. This result may be because *Gossypium hirsutum* is tetraploid and has a doubled genome compared to the diploid organisms *Gossypium raimondii* and *Gossypium aridum*. The difference in the number of these types of genes identified by Dou et al. from *Gossypium hirsutum* and the number that we found might be present because these genes were cloned by Dou et al. from *Gossypium hirsutum* using a homologous cloning method based on *Gossypium raimondii*, which led to the loss of some genes (Dou et al., 2014). In the evolutionary analysis, it was found that members within the same clade shared a similar motif composition, indicating their close evolutionary relationship. In addition, the group IId *GhWRKY* genes shared similar exon-intron patterns, which may be the result of a series of gene duplication events (Guo et al., 2014). The close relationship among group IId WRKY genes suggested that these genes have experienced similar evolutionary events (Li et al., 2017b).

Gene duplication events occurred among group IId WRKY genes. Gene duplication events can be divided into two categories, namely, tandem, and segmental duplication. The distribution of two or more genes on the same chromosome is defined as a tandem duplication, while a distribution of these genes on different chromosomes is considered segmental duplication (Liu et al., 2011). Fifteen gene pairs were identified to be involved in segmental gene duplication events. Gene duplication events were one of the main contributors to evolutionary dynamics, and they had a significant effect on genomic rearrangements and expansions (Ohno et al., 1968; Vision et al., 2000; Chothia et al., 2003). Therefore, the occurrence of segmental duplications contributed to the expansion of the group IId WRKY subfamily. However, no tandem duplications were detected, indicating that the segmental duplication event was the major driving force that led to the expansion of the group IId WRKY genes in the evolution process (He et al., 2016).

The Ka/Ks values of the 15 gene pairs were calculated to identify the selective pressure acting on these homologous protein-coding gene pairs at the protein sequence level. The Ka/Ks values of 14 gene pairs were lower than 1, indicating that these gene pairs are undergoing purification selection and tend to eliminate deleterious mutations during evolution. The Ka/Ks value of one gene pair was >1, indicating that its members are under positive selection, thereby accelerating the fixation of beneficial mutations (Hurst, 2002; Ling et al., 2011).

Through expression pattern analysis of the group IId WRKY genes, it was found that segmental duplicate gene pairs exhibited similar expression trends in different tissues and under drought and salt treatments. Gene orthology predicts similar functional roles (Zhang and Wang, 2005). Therefore, duplicate gene pairs may play vital roles in adapting to external environments during evolution and maintaining the stability of the genetic system when it is attacked by environmental stimuli (Gu, 2003; Chapman et al., 2006). However, studies have shown that expression divergence between duplicated WRKY genes is also present, but the reasons for the difference in expression remain unclear (Ramamoorthy et al., 2008).

We analyzed the expression of group IId WRKY genes in 10 different tissues. The results demonstrated variation in the expression levels of these genes. Most group IId WRKY genes were highly expressed in stems, which is consistent with observations in sesame (Li et al., 2017a). In total, 15 genes were highly expressed in stem, 5 were highly expressed in petal, and 4 were highly expressed in torus. Meanwhile, 5 genes were highly expressed in all tissues. The genes with high expression levels in a specific tissue may play roles in plant growth and development (Ramamoorthy et al., 2008). Therefore, we concluded that the highly expressed genes or tissue-specific genes may play an important regulatory role in cotton development, and further research is needed to verify their functional roles.

Evidence is accumulating that abiotic stresses such as drought and high salinity have adverse effects on plant growth and development. Under abiotic stresses, stress-responsive genes are induced to adapt to various developmental and physiological changes (Karanja et al., 2017). Abiotic stresses can also induce the expression of WRKY TFs (including group IId WRKY TFs) and activate signal transduction networks to increase stress resistance in plants (Schluttenhofer and Yuan, 2015). Previous reports showed that at least 7 group IId WRKY genes in moso bamboo (Li et al., 2017b) and 6 in *Cucumis sativus* (Ling et al., 2011) are differentially expressed under abiotic stresses. In our study, the majority of the group IId WRKY genes exhibited upregulated expression patterns in response to drought and salt stresses, indicating their important roles in abiotic stress responses (Xiong et al., 2013). In addition, the Gh_A11G1801 gene showed similar expression patterns with its corresponding gene GhWRKY42 under both drought and salt conditions (Gu et al., 2018b) using different primers, indicating the reliability of our results. In *Arabidopsis, AtWRKY15* was involved in the osmotic stress response (Vanderauwera et al., 2012). Gh_D11G1963, an ortholog of *AtWRKY15*, was highly upregulated under drought and salt treatment. *AtWRKY11* participated in abiotic stress (Ali et al., 2018), and Gh_A06G2118, an ortholog of *AtWRKY11*, was also highly elevated by abiotic stress. These results indicated that some WRKY orthologs are functionally conserved across different plant species. In addition, a group IId WRKY gene, *GhWRKY17*, with 99.37% similarity to Gh_A06G0179, was differentially induced by PEG6000 and salt treatment, and overexpression of *GhWRKY17* reduced tolerance to drought and salt stresses in transgenic *Nicotiana benthamiana* (Yan et al., 2014). *GhWRKY39* (Gh_Sca005611G02, 99.67% identity) was induced by salt treatment, and overexpression of *GhWRKY39* conferred enhanced tolerance to salt treatment in transgenic *N. benthamiana* (Shi et al., 2014). Similarly, our data showed that Gh_A11G1801 was strongly elevated by drought stress. GUS activity was enhanced in transgenic *Arabidopsis* plants with the Gh_A11G1801 promoter under drought treatment, and silencing of Gh_A11G1801 decreased drought tolerance in cotton, demonstrating that Gh_A11G1801 plays an important role in the cotton response to drought. Therefore, the expression patterns of the group IId WRKY genes may contribute to a more thorough understanding of their specific functions in cotton.

MDA is the final decomposition product of lipid peroxidation and can reflect damage to the plant membrane system and the resistance of the plants (Yoshimura et al., 2004). Our results showed that the VIGS plants accumulated more MDA under drought treatment than non-VIGS plants, indicating that the VIGS plants promote lipid oxidation. Drought could cause the accumulation of reactive oxygen species (ROS) and ROS-related damage (Krasensky and Jonak, 2012). Hydrogen peroxide (H_2_O_2_) had emerged as one of the prominent ROS species that participate in stress signals and oxidative damage (Yoshioka et al., 2003; Laloi et al., 2004). Excessive production of H_2_O_2_ can cause leaf cell death, thus leading to leaf necrosis in plants (Zheng et al., 2006). The CAT enzyme, an ROS-scavenging enzyme, plays an important role in regulating intracellular H_2_O_2_ levels. The Gh_A11G1801 VIGS plants displayed less CAT activity than the CK plants when challenged with water-withholding treatment. The reduced CAT antioxidant enzyme activities might reflect the decreased ability to scavenge H_2_O_2_ and an enhancement of oxidative injury in VIGS plants under drought stress. These results suggest that silencing Gh_A11G1801 affects the antioxidant system and that Gh_A11G1801 may be involved in the ROS-mediated signaling network in cotton.

In summary, a systematic analysis of the group IId WRKY genes was performed in cotton. Evolutionary analysis revealed their similarity and conservation in their gene structure and function. Through expression analysis, genes whose expression was tissue-specific and/or induced by drought or salt were identified. The Gh_A11G1801 gene was endogenously silenced via VIGS, and the VIGS cotton plants exhibited decreased tolerance to drought treatment. Further analysis will be necessary to provide new insights into the biological roles of the group IId WRKY genes in cotton.

## Author contributions

SY, HaW, and HeW designed the experiments. HS, LL, PC, and CZ collected the sequences. LG performed the experiments and wrote the manuscript. ME, ZS, LM, and CW revised the language. All the authors read and approved the final manuscript.

### Conflict of interest statement

The authors declare that the research was conducted in the absence of any commercial or financial relationships that could be construed as a potential conflict of interest.

## Abbreviations

ABA: abscisic acid
CAT: catalase
ET: ethylene
GUS: β-glucuronidase
JA: jasmonic acid
MDA: malondialdehyde
PEG: polyethylene glycol
ROS: reactive oxygen species
SA: salicylic acid
SEs: standard errors
TFs: transcription factors
VIGS: virus-induced gene silencing.
