# Supplementary material for: Identification, Expression, and Functional Analysis of the Group IId WRKY Subfamily in Upland Cotton (Gossypium hirsutum L.)
Source: Front Plant Sci. 2018 Nov 21;9:1684. doi: 10.3389/fpls.2018.01684 (PMC6259137; doi:10.3389/fpls.2018.01684)
Supplement: Supplementary file 1 [file Table_1.docx]

Table S1. Primers used in this study

| **Primer name** | **Sequence (5’- 3’)** | **Description** |
| --- | --- | --- |
| *GhActin*-F | ATCCTCCGTCTTGACCTTG | Primers used in qRT-PCR |
| *GhActin*-R | TGTCCGTCAGGCAACTCAT |  |
| Gh_A02G1488-F | CCGCTTCTGGTCTCGAAAGTGT |  |
| Gh_A02G1488-R | TCCATGGAGGATCTCGTCGGAG |  |
| Gh_A06G2118-F | ATCTCAACGCCGGCGAACATAA |  |
| Gh_A06G2118-R | AAACTCTGCGGCTGCGATTGAA |  |
| Gh_A07G0017-F | CCGACATTCCGGCAGACGAATA |  |
| Gh_A07G0017-R | GGGACACCCTCTGACCGTACTA |  |
| Gh_A11G0997-F | CCCTAAAGAACCCCAAACCAC |  |
| Gh_A11G0997-R | ATCTTCATCTGTACTGCTCGTT |  |
| Gh_A11G1801-F | TGTAAGAGGATGTCCAGCCCGT |  |
| Gh_A11G1801-R | ATAGGGAATGGTTGTGCTCGCC |  |
| Gh_D03G0226-F | GAAACCGCCGTTCAAGAAGCAG |  |
| Gh_D03G0226-R | GGGCGGTGACTTTACAGTCCATT |  |
| Gh_D07G0023-F | TTAACAGAACCGGTCACGCTCG |  |
| Gh_D07G0023-R | AGCAGCGGATGATGACGATGAG |  |
| Gh_D11G1141-F | CCCATGACACACTGGGGTTTCT |  |
| Gh_D11G1141-R | TCATCCCAAAACTTGCTTCGCA |  |
| Gh_D11G1963-F | AGAGGATGTCCAGCTCGCAAAC |  |
| Gh_D11G1963-R | ATAGGGAATGGTTGTGCTCGCC |  |
| Gh_Sca005611G01-F | GTATGAGAGGTTGCCCTGCGAG |  |
| Gh_Sca005611G01-R | GCGATGGTAACCTTGGGTGGTT |  |
| AtActin2-F | AAGCTCTCCTTTGTTGCTGTT |  |
| AtActin2-R | GACTTCTGGGCATCTGAATCT |  |
| GUS-F | TCAGTGGCAGTGAAGGG |  |
| GUS-R | GAGGTACGGTAGGAGTTGG |  |
| Gh_A11G1801-XbaI -F | CTAGTCTAGACTGTTCAACCCGATCAAGAG | VIGS |
| Gh_A11G1801-BamHI -R | CGCGGATCCGGAAGACCCACTAGTACA |  |
